# Leveraging innovative technology and health data to enhance access to emergency care and referral services in Kenya

**DOI:** 10.1093/oodh/oqaf019

**Published:** 2025-08-22

**Authors:** Daniela Pugsley Reis, Pamela Nawaggi, Anna Fraenzel, Caitlin Dolkart

**Affiliations:** Rescue Global, Nairobi-Kenya Planning House, Lower Kabete Road, P.O. Box 2578-00606 Nairobi Kenya; Rescue Global, Nairobi-Kenya Planning House, Lower Kabete Road, P.O. Box 2578-00606 Nairobi Kenya; Rescue Global, Nairobi-Kenya Planning House, Lower Kabete Road, P.O. Box 2578-00606 Nairobi Kenya; Rescue Global, Nairobi-Kenya Planning House, Lower Kabete Road, P.O. Box 2578-00606 Nairobi Kenya

**Keywords:** emergency care, LMICs, digital health, fleet coordination, data-driven operations, Kenya

## Abstract

**Background:**

Access to timely emergency medical services is a persistent challenge in low- and middle-income countries, where systems are often fragmented and under-resourced. In Kenya, gaps in centralized dispatch, ambulance coverage and coordination have led to prolonged emergency response times.

**Methods:**

Rescue.co’s proprietary Flare platform and services were implemented in urban and rural Kenya, and expanded to Uganda and Tanzania. This case examined operational data, dispatch records and platform iterations in Kenya from 2017 to 2025.

**Implementation:**

Rescue.co uses a proprietary tech stack—including global positioning system (GPS), Android devices, Google Maps application programming interface, and telecom tools—to enable real-time fleet coordination and route optimization. A 24/7 dispatch center staffed by trained personnel manages end-to-end response, using dispatch data to refine system performance and scalability.

**Outcomes:**

Rescue.co reduced average response times from >160 minutes to 13 minutes in urban areas and <30 minutes in rural regions in Kenya. By 2025, 47 000+ emergency responses had been coordinated, 800+ ambulance providers had been connected to the network, and 2000 health facilities had been linked.

**Conclusion:**

Rescue.co illustrates how a locally developed, scalable digital solution has transformed emergency medical services delivery in Kenya, with the potential to be scaled to other low- and middle-income countries. Its experience offers practical insights for health system leaders and policymakers advancing digital emergency care.

## INTRODUCTION

Access to timely emergency medical services (EMS) is critical for reducing morbidity and mortality, yet remains highly inadequate across many low- and middle-income countries (LMICs) [1]. EMS systems in these settings are often fragmented, underfunded and poorly coordinated [2]. The World Health Organization (WHO) has highlighted that a significant proportion of preventable deaths could be averted through improved access to emergency care [3]. Provision of EMS in Kenya is a devolved function of the 47 counties as per the Constitution of Kenya 2010. The services are mainly offered by hospital-based ambulance services run by the county health departments. These services are complemented by nonprofit agencies and private providers. Despite the progress made in the EMS sector over the years, the current EMS system in Kenya faces many challenges with regard to funding, management, workforce and infrastructure, often leading to inadequate access and inefficiency of the service. Despite efforts to expand healthcare coverage through both public and private sectors, the emergency response infrastructure remains underdeveloped; >70% of Kenyans live in rural or peri-urban settings, where infrastructure gaps are acute [4].

Digital health innovations have emerged as promising tools to modernize health systems and improve system efficiencies in LMICs, particularly through the use of real-time data and mobile technology. Solutions such as cloud-based dispatch platforms, GPS tracking and automated triage systems offer a pathway to improve ambulance allocation and case prioritization in low-resource environments [5].

In line with this global momentum, various regional strategies—including the African Union Digital Health Strategy 2020–2030 and WHO’s global digital health frameworks—call for investments in integrated health information systems and cross-sector collaboration to strengthen emergency response [6].

This case study explores the development and implementation of Rescue.co, a Kenya-based digital EMS coordination platform that centralizes ambulance dispatch, coordinates public and private providers and optimizes care through real-time communication between ambulances and hospitals. Utilizing GPS-enabled devices, automated routing and a 24/7 dispatch center staffed by trained triage personnel, the platform offers a flexible model of digital innovation, tailored to the infrastructure and health priorities of LMICs. By capturing Rescue.co’s evolution and outcomes, this case provides actionable insights for countries aiming to strengthen EMS systems through digital transformation.

## CASE DESCRIPTION

Access to emergency care may be hindered by fragmented ambulance networks, poor referral coordination, insufficient human resources and unequal distribution of healthcare facilities [7]. Many African countries lack centralized EMS protocols or dispatch systems, leaving patients reliant on ad hoc arrangements or informal transport in emergencies. Urban centers, while better resourced, face their own obstacles, such as persistent traffic congestion, limited traffic prioritization for ambulances and an absence of emergency vehicle lanes [8]. In rural areas, the absence of paved roads, long distances to facilities and limited ambulance coverage pose even greater barriers [9].

In Kenya, the EMS landscape exemplifies these structural challenges. With a population exceeding 53 million, the country lacks centralized dispatch infrastructure, the national emergency hotline is not optimized and standard referral protocols are not fully implemented [10]. In urban areas such as Nairobi, severe traffic congestion and a patchwork of uncoordinated ambulance fleets contribute to prolonged delays in response. Prior to 2017, the average ambulance response time was estimated to be 162 minutes [5]. In rural settings, delays could last several hours or even days, often forcing families to resort to taxis, motorcycles or private cars for emergency transport, if even available [4, 11]. These delays have significant consequences for time-sensitive conditions such as postpartum hemorrhage, stroke, trauma and neonatal distress, contributing to avoidable morbidity and mortality [12].

**Figure 1:**
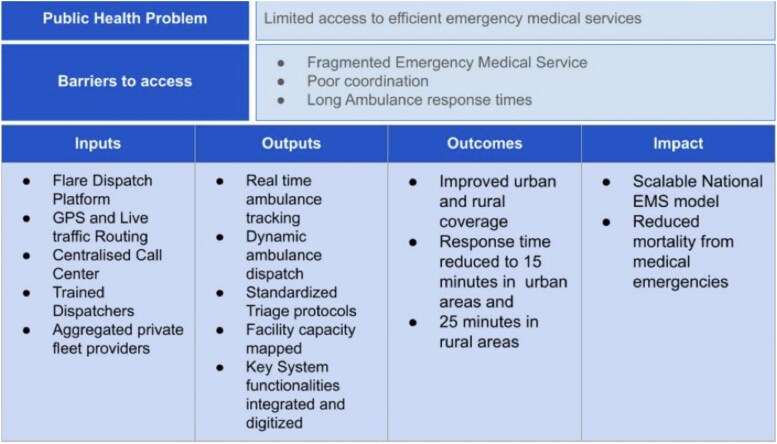
Theory of change for the launch of Rescue.co’s digital EMS coordination platform

To address critical gaps in Kenya’s emergency response landscape, and specifically to address the challenge of long response times, Rescue.co was launched in 2017 as a digital EMS coordination platform (Fig. 1).

The company leverages its proprietary technology platform called Flare, which is an integrated, technology-driven system. The system was designed to aggregate a fragmented fleet of individual private ambulance providers and map health facility capacities. These are visualized on the Flare platform, allowing a dispatcher to send the closest appropriate ambulance to the scene of an emergency, and transfer a patient to the closest adequately equipped facility for the specific medical case. The platform was designed with the intention to reduce the average ambulance response time in Kenya from 162 minutes in 2017 [5] to a target of 15 minutes in urban locations, and 25 minutes in rural areas.

**Figure 2:**
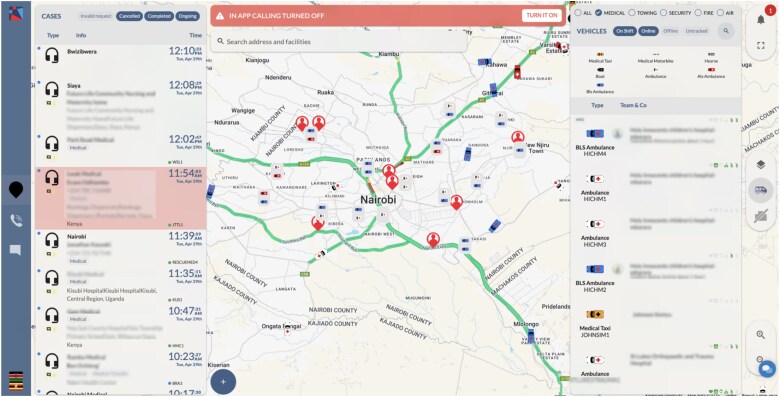
A screenshot of Rescue.co’s proprietary dispatch platform, flare dispatch’s interface showing active emergency cases, their location and available ambulances

Flare Dispatch (Fig. 2), is a proprietary software suite that enables real-time GPS tracking, cloud-based dispatch operations and standardized clinical triage. Ambulances participating in the network are equipped with android-enabled devices and connected to a centralized command center staffed by trained dispatchers who oversee case triage and vehicle allocation. The system leverages Google Maps application programming interfaces (APIs) to dynamically route ambulances based on traffic conditions, proximity and urgency. To accommodate varying levels of digital access, emergency requests can also be initiated through a toll-free line, mobile app and short message service (SMS).

**Figure 3:**
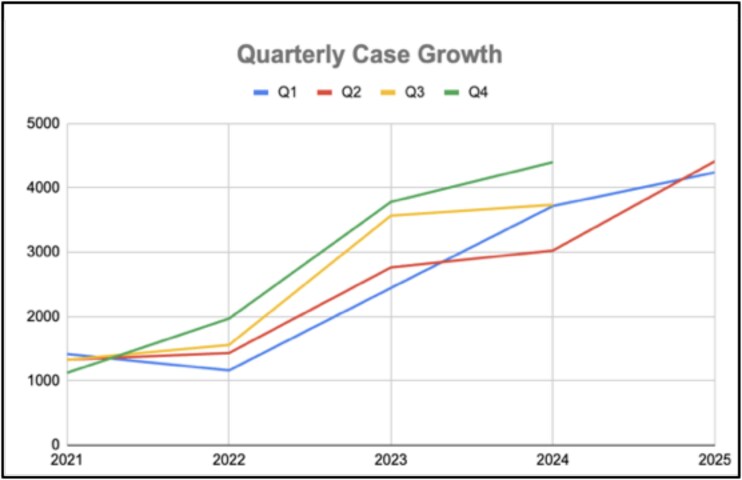
Quarterly case growth in Kenya

**Figure 4:**
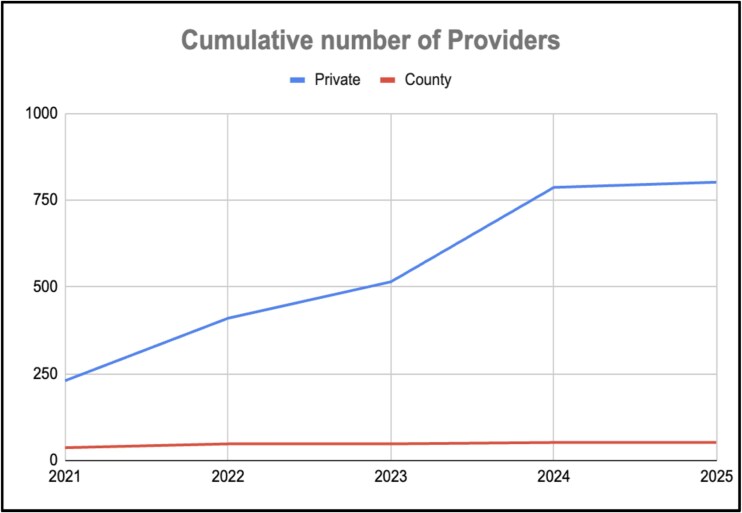
Cumulative number of providers

By 2025, Rescue.co had onboarded >800 public and private ambulance providers and coordinated >47 000 emergency responses across all 47 counties in Kenya, transforming access to care at both urban and rural levels (Figs 3 and 4). Crucially, the platform enforces dispatch neutrality, allocating ambulances based solely on response time and clinical need and capability, rather than provider affiliation, effectively dismantling the siloed, facility-bound EMS model that previously limited equitable access [5, 6].

**Figure 5:**
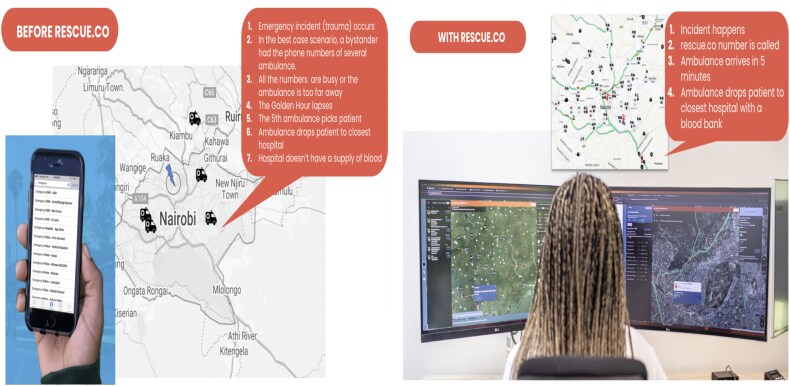
Typical experience accessing Kenya’s emergency transport system in the case of an emergency (for example: traumatic injury) before and after Rescue.co

The platform also features hospital integration modules that provide real-time alerts to receiving facilities about incoming patients, allowing clinical teams to mobilize resources and prepare for arrival. This closed-loop system improves care continuity and enhances the responsiveness of the broader health system (Fig. 5).

Rescue.co’s development and expansion were facilitated through deliberate and sustained engagement with a wide range of external stakeholders, each playing a distinct role in the platform’s design, execution and sustainability. Ambulance providers, including public, private and NGO-operated fleets, were incorporated through formal service-level agreements. These partnerships included the provision of dispatch technology, GPS devices and training in triage protocols, enabling even under-resourced providers to participate in a coordinated national EMS system. Health facilities, particularly county referral hospitals, were linked to the dispatch platform through hospital alert systems and dashboard interfaces. This integration allowed clinicians to receive timely updates about incoming patients, improving communication and readiness on the ground. Feedback from regular engagement sessions with clinical teams pointed to notable improvements in coordination, reduced delays and better continuity of care.

Rescue.co also collaborated closely with policymakers and public health authorities, particularly the Ministry of Health and county health departments, such as those in Nairobi and Siaya to ensure alignment with Kenya’s Digital Health Strategy (2016–2025), national digital health policies and contribution to universal health coverage (UHC) goals [13]. The platform’s operational data further supported government efforts in planning and optimizing EMS policy, resource distribution and service delivery strategies.

Additionally, the intervention included engagement of a wide range of corporate and institutional clients—including private employers, educational institutions, NGOs and multilateral agencies—who adopted Rescue.co’s service as a health and safety benefit for employees and beneficiaries. These partners received tailored onboarding and ongoing support through Rescue.co’s Member Success and Sales Training teams, ensuring high service utilization and satisfaction. Health insurance providers also played a crucial role by integrating Rescue.co into both private and micro-insurance products. This reduced financial barriers for patients while supporting the platform’s sustainability through diversified revenue streams. Finally, Rescue.co’s functionality was underpinned by collaborations with leading technology firms, including Google Maps API and Amazon Web Services, which enabled real-time vehicle tracking, cloud-based communication infrastructure and predictive fleet analytics. These capabilities allowed the dispatch center to monitor system-wide utilization, identify bottlenecks and allocate resources efficiently.

Together, these coordinated efforts have established a scalable, data-driven emergency response ecosystem capable of addressing critical public health challenges in LMICs. Rescue.co exemplifies how locally led digital health innovation, rooted in systems thinking and enabled through cross-sector collaboration, can improve efficiency of emergency care delivery in settings with fragmented health infrastructure.

Beyond traditional EMS, Rescue.co has introduced targeted interventions that respond to other critical public health and safety concerns and diversify revenue streams. Its Roadside Rescue service addresses Kenya’s rising burden of road traffic injuries—one of the leading causes of death—by offering a combined product providing both on-site medical and mechanical assistance at the scene of vehicle incidents, using a combination of ambulance and tow trucks and mechanics. In parallel, security response services, available in select urban areas, provide rapid deployment of vetted security personnel to escort and protect members in situations where they feel concerned about their safety, mobility or access to care.

Rescue.co’s AirEvac program enhances access to emergency care in remote areas of Kenya, Rwanda, Uganda and Tanzania by providing medical (MEDEVAC) and non-medical (CASEVAC) air evacuations to residents and also to tourists visiting Kenya who typically travel to remote locations which are challenging to cover with ground ambulance services alone. It supports critically ill patients needing urgent transport to specialized facilities and helps improve regional emergency coordination.

Rescue.co’s services reflect a public health-focused approach, addressing critical issues like emergency delays, trauma, referral gaps and care inequities through a digital, data-driven model.

## IMPLEMENTATION

The implementation of Rescue.co’s digital EMS platform unfolded in a phased, iterative process from 2015 through 2025. Each phase was shaped by continuous stakeholder engagement, operational feedback and adaptation to evolving healthcare landscapes. The process combined software development, integration with emergency transport providers and health facilities and targeted training to ensure functionality in both urban and rural contexts. What began as a Nairobi-based pilot has since become a national EMS coordination system, now expanding across borders and into new technological frontiers.

### Phase 1: research and early prototyping (2015–2016)

Founded in 2015, Flare Emergency Response Ltd. began with extensive field research involving emergency responders, hospitals and ambulance operators to identify systemic barriers to timely care. The team developed early prototypes of the Flare Dispatch platform, focusing on GPS tracking, digital triage and ambulance location mapping. Basic workflows were tested using publicly available APIs and SMS tools to assess feasibility in low-resource settings.

### Phase 2: official launch and minimum viable product deployment (2017)

In 2017, Rescue.co launched its minimum viable product (MVP), linking a small network of vetted ambulance providers to a centralized dispatch center. The system included Google Maps routing, a cloud-based dispatch dashboard and Android-enabled tracking devices in ambulances. Operating from a shared workspace in Nairobi, a small team of trained dispatchers provided 24/7 coverage. During this phase, real-time feedback helped refine triage protocols and user interactions.

### Phase 3: platform optimization and growth (2018–2019)

With rising demand, Rescue.co integrated custom case management tools. Dispatch workflows were standardized and enhanced with clinical triage protocols. The provider network grew through partnerships with private ambulance companies, hospitals, insurers and employers. Subscription-based services were introduced, and the platform expanded beyond Nairobi, handling hundreds of cases each month.

### Phase 4: national scale, COVID-19 response and major institutional collaborations (2020–2022)

The COVID-19 pandemic accelerated platform growth and tested system resilience. Rescue.co supported government pandemic logistics and secured its first large-scale public sector contract, enabling EMS coordination across counties. Partnerships with MSD for Mothers [14], USAID, and Jacaranda Health led to critical initiatives in maternal health and trauma care. These included free ambulance services for obstetric emergencies and research on injury trends and hospital readiness, informed by Rescue.co’s real-time clinical data. The platform onboarded hundreds of new ambulance providers, doubled the dispatch staff and expanded coverage to all 47 Kenyan counties. This phase marked Rescue.co’s emergence as a national EMS backbone and demonstrated its value as a public-private partner in health system strengthening.

### Phase 5: product diversification and regional expansion (2023–2024)

Rescue.co diversified its offerings to address other public health and safety gaps. Roadside by Rescue.co was launched to respond to rising road traffic injuries and the Safe Travels product for AirEvac services was introduced to coordinate medical evacuations, including cross-border transfers. The dispatch platform was upgraded to manage both ground and air logistics, with clinical decision trees for MEDEVAC and CASEVAC. Rescue.co also expanded services to institutional clients—including NGOs, corporates and schools—supported by dedicated onboarding teams. In 2024, EMS operations formally launched in Uganda and Tanzania, integrating local ambulance providers and hospitals while adapting triage protocols to new regulatory environments.

### Phase 6: strategic innovation and artificial intelligence integration (2025–ongoing)

By 2025, Rescue.co had coordinated over 47 000 emergency cases, integrated more than 800 providers, and mapped over 2000 healthcare facilities across its service areas As part of its future strategy, Rescue.co plans to introduce artificial intelligence (AI) into dispatch, triage and system planning. Proposed applications include predictive dispatching, automated triage and demand forecasting, using historical response data to enhance efficiency and decision-making. To support this, Rescue.co intends to partner with academic and technical experts to ensure safe, ethical and context-appropriate AI development. Planned investments in data infrastructure and clinical coding will enable model training and integration. This initiative reflects Rescue.co’s long-term vision to build a more intelligent, responsive and equitable EMS system.

Rescue.co’s experience offers valuable lessons for digital health deployments in LMICs. Key enablers included early stakeholder alignment, iterative development grounded in user feedback and flexibility to integrate with both public and private systems. The phased approach—from MVP to full-scale national implementation—allowed for adaptation, validation and learning at each stage. Rescue.co’s ability to embed core technologies such as GPS routing, digital triage and dispatch neutrality within local workflows underscores the importance of contextual design. Its partnerships with governments, donors and technology firms further highlight how cross-sector collaboration can drive sustainable, high-impact health system transformation.

## OUTCOMES AND IMPACT

**Figure 6:**
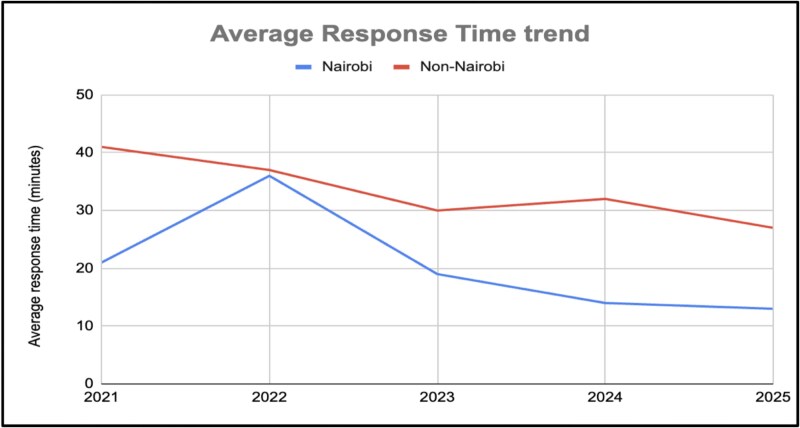
Trend of average response time in Nairobi and rural counties

This case study does not present a formal clinical or statistical evaluation. Rather, its findings are based on internal operational data and dispatch records. Since launching in 2017, Rescue.co has significantly improved EMS delivery across the country. The most immediate and transformative change has been the reduction in ambulance response times. In Nairobi and other urban centers, response times dropped from >160 minutes [5] to <15 minutes (Fig. 6). In rural counties, previously characterized by multi-hour or multi-day delays, response times were reduced to <30 minutes. These improvements ensure that patients reach appropriate emergency care within globally-recognized response times. Now the focus is towards evaluating the impact of these improved response times and referral pathways on patient outcomes, particularly for trauma cases, maternal and neonatal cases.

**Figure 7:**
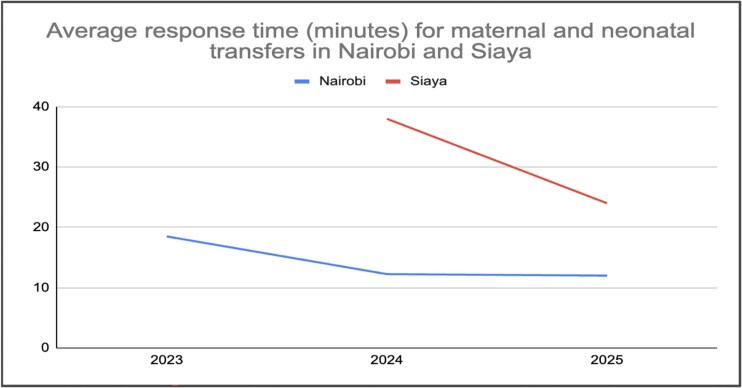
Trend of average response time for maternal and neonatal emergency transfers in Nairobi and Siaya county

Notably, Rescue.co has deployed donor-funded maternal health initiatives to support emergency response for Emergency Obstetric and Newborn Care (EMONC) complications such as postpartum hemorrhage and fetal distress. Facilities participating in these programs, in Nairobi, Kakamega and Siaya counties, reported faster ambulance turnaround times (Fig. 7), and the platform embedded maternal prioritization into dispatcher workflows and clinical training. Pre-alert systems allowed hospitals to prepare for critical cases, with the aim of improving maternal care outcomes and reducing delays in intervention.

Despite these successes, implementation challenges persisted. Early provider resistance centered on concerns about data sharing, competition and adopting new workflows. In remote regions, poor mobile network coverage compromised GPS reliability and real-time coordination.

The platform has reshaped how emergency care is coordinated and delivered in Kenya, replacing fragmented systems [8, 12, 15, 16] and leveraging the private sector with real-time, data-driven workflows that improve response times and resource allocation.

These lessons are relevant for LMICs seeking to optimize fragmented EMS systems. While future evaluations should include rigorous clinical outcome measures, Rescue.co demonstrates the real-world value of digital EMS platforms when designed for local conditions and sustained through continuous engagement and system adaptation.

## DISCUSSION

Rescue.co’s experience in Kenya demonstrates the potential of digital coordination platforms to address long-standing inefficiencies in EMS in LMICs. While the platform’s operational impact in reducing response times and improving system integration is evident, its broader significance lies in offering a replicable model for health system innovation that is both context-aware and technologically adaptable.

Across many LMICs, EMS systems remain fragmented, under-regulated and unevenly distributed. Studies from countries such as Nigeria, Bangladesh and India highlight similar barriers to timely emergency care—namely, poorly coordinated transport systems, lack of trained dispatchers, insufficient data visibility and weak integration between pre-hospital and in-hospital care [17]. In these contexts, digitized dispatch platforms have shown promise. For example, India’s 108 Emergency Response Service has demonstrated reductions in response times and improved maternal outcomes through centralized coordination [16, 18, 19]. However, these programs often operate under government mandate with limited integration across private sector actors, a gap that Rescue.co addresses through its public-private-neutral model.

Like in Kenya before the advent of the Rescue.co; the state of EMS in Uganda and Tanzania is critical. In Tanzania, despite tremendous improvement in health care delivery over the past decades, emergency-related deaths due to non-communicable diseases and injuries have increased, while infections, maternal- and infant-related emergencies still remain an important cause of mortality in the country [20]. However, there is no public EMS system, and the private ambulances that do exist are prohibitively expensive for average civilians [21]. In Uganda, getting to a hospital in a medical emergency is challenging, with <50% of patients reached by an ambulance within an hour (the ‘Golden Hour’) [22], increasing mortality and serious and long term health problems. In addition, the country has limited resources with only 240 government-owned ambulances, far below the 460 needed, and private ambulance numbers remain unclear [22]. Patients often struggle to find an available ambulance due to an inefficient dispatch system and unreliable emergency numbers, and 95% of Ugandans do not know an emergency number [23].

**Figure 8:**
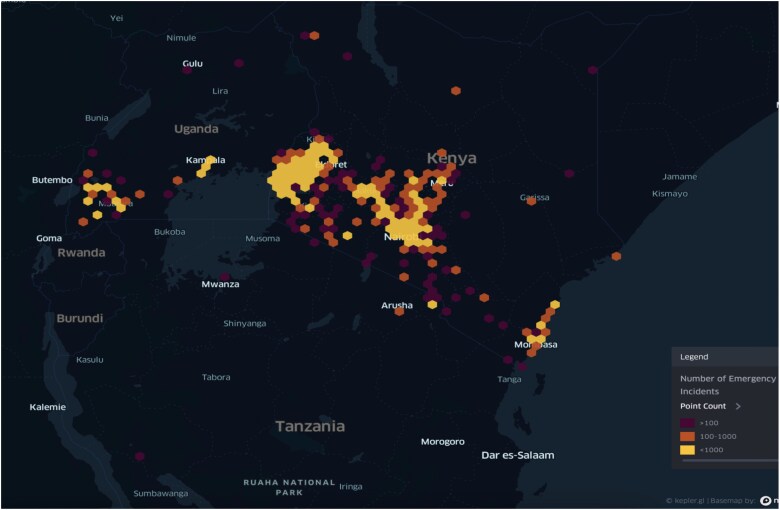
A heatmap showing Rescue.co’s emergency response reach within East Africa

By the end of 2024, Rescue.co had formally expanded its EMS operations to Uganda and Tanzania, marking a significant milestone in regional replication. The rollout involved the coordination of ambulance providers and referral hospitals, resulting in the integration in 21 districts in Uganda, with over 20 ambulances and the management of over 400 emergency cases in the first quarter of operations. In Tanzania, the rollout involved 8 districts, and 20 cases in the same period (Fig. 8). While the expansion required adaptations to local health conditions—such as revising triage protocols, onboarding new partners and navigating regulatory environments—the platform’s core functionalities, including GPS-based routing, digital triage and dispatch neutrality, proved robust and effective. This experience underscored the platform’s flexibility and potential to scale across diverse LMIC contexts.

Rescue.co’s strategic expansion encompasses both service breadth and geographic reach. To reduce preventable mortality linked to delayed referrals and inadequate transport, the platform also offers non-emergency medical transfers, supporting chronic care, interfacility referrals and hospital discharges. The expansion to Uganda and Tanzania introduced new regulatory and contextual complexities. Adapting the platform required revising triage protocols, onboarding local stakeholders and navigating health policy differences. These challenges were addressed through the same iterative and collaborative approach that has proven effective in Kenya. However, constraints in funding and human resources limited the speed of expansion and the ability to establish more formalized evaluation frameworks.

A key consideration for scaling Rescue.co’s model in different countries lies in understanding and adapting to the unique operational and cultural landscapes of each setting. This includes engaging with local stakeholders early to align with existing emergency response protocols and workflows, customizing technological tools to reflect infrastructure capacity (such as variable mobile or internet coverage), and ensuring compliance with national data protection and health regulation frameworks. Moreover, building trust with both public and private ambulance providers is essential to foster buy-in, dismantle competitive silos and ensure comprehensive geographic coverage.

Rescue.co’s ability to embed itself within Kenya’s health ecosystem was enabled by its flexibility in technology and protocol design, its emphasis on stakeholder engagement, alignment with national policies and its commitment to neutrality in dispatch. These qualities must remain central in any cross-country adaptation. While these adaptations presented challenges in Uganda and Tanzania, they also reinforced the model’s modularity and its capacity to serve as a foundational infrastructure layer for emergency response across diverse geographies. Its success in Uganda, Tanzania and broader East Africa shows strong potential for expansion in LMICs with increasing mobile connectivity and evolving health systems. Rescue.co has demonstrated its value in strengthening regional health networks and cross-border emergency coordination, especially where traditional EMS systems are lacking.

Future scale-up will also benefit from Rescue.co’s existing partnerships with corporate and institutional clients that operate regionally. These clients—ranging from multinational NGOs to health insurers and employers—can serve as anchor partners in new markets, offering both a user base and contextual knowledge to accelerate onboarding and localization. Their presence across East Africa and beyond presents a strategic opportunity to align EMS platform expansion with occupational health priorities and existing care networks.

In terms of global digital health strategy, Rescue.co’s model aligns with the WHO’s recommendations for strengthening emergency care systems through digital innovation, multisectoral collaboration and the use of real-time data for system responsiveness [24]. It also complements the African Union’s Digital Health Strategy 2020–2030, which advocates for interoperable health information systems, public-private partnerships and locally led technology solutions [25]. In contrast to top-down, donor-driven digital health implementation that often falters due to a lack of local ownership, Rescue.co offers an example of bottom-up innovation that is scaled nationally through system co-creation and continuous iteration.

Nevertheless, the absence of formal clinical impact evaluations remains a limitation that should be addressed in future research. Studies are currently underway to establish impact on maternal mortality and survival after trauma One of the studies is measuring the impact of the Rescue.co centralized emergency service response system on mortality and morbidity for emergency trauma patients in Kenya and will determine if trauma patients using Rescue.co EMS experience different morbidity and mortality outcomes compared to a control group of trauma patients who do not use Rescue.co services.

While the operational improvements—such as faster response times and improved provider coordination—are important indicators, measuring patient outcomes (e.g. mortality, morbidity and time to definitive care) is essential to build a more robust evidence base and inform health policy adoption. Comparative research with other EMS reforms and models, such as Rwanda’s Service d’Aide Medicale d’urgence system or Tanzania’s m-mama, could also offer valuable benchmarks for evaluating Rescue.co’s long-term contribution to population health outcomes.

In summary, Rescue.co’s experience demonstrates that digital health platforms can meaningfully improve emergency care delivery in LMICs when grounded in local realities, supported by stakeholder trust and adaptable to institutional frameworks. For other countries seeking to modernize EMS in fragmented, under-resourced environments, this case offers both a proof of concept and a practical roadmap. Success, however, will depend on more than just technology. It will require careful contextualization, long-term investment in partnerships and sustained attention to the systemic, social and infrastructural variables that shape emergency response delivery across diverse settings.

## ETHICAL AND DATA GOVERNANCE CONSIDERATIONS

This study utilized operational data from Rescue.co’s digital emergency response platform to assess improvements in emergency medical service efficiency in Kenya. This review did not involve direct interaction with human participants, and no personally identifiable information (PII) was accessed or reported.

Rescue.co operates under a strict privacy policy, which governs the collection, processing and storage of user data, including personal and location-based information. The platform ensures data security through encryption and compliance with international data protection standards. [26–28].

## CONCLUSION

Rescue.co’s experience demonstrates the transformative potential of digital platforms to address critical gaps in EMS in Kenya, and the potential for this scalable model to be transplanted to other LMICs—currently Tanzania and Uganda. By centralizing ambulance dispatch, integrating diverse providers and enabling real-time coordination with hospitals, Rescue.co improved response times, reduced redundancies and enhanced access to timely appropriate care, particularly for maternal and trauma emergencies. Its success underscores the importance of stakeholder collaboration, flexible technology design and continuous adaptation to local operational realities.

For countries seeking to strengthen EMS systems, this case highlights several practical considerations: digital interventions must align with existing health infrastructure, foster trust among providers and be embedded within broader health system strategies. Governments and health planners should prioritize regulatory environments that support public–private integration and invest in digital readiness, including connectivity and data governance. Regional replication efforts can be accelerated through strategic partnerships with cross-border organizations and clients operating in multiple markets.

Future work should include rigorous impact evaluations to assess patient-level outcomes, cost-effectiveness and long-term system resilience. As digital health continues to evolve, Rescue.co offers a compelling example of how locally led, data-driven innovation can address entrenched public health challenges and support the realization of UHC through scalable, sustainable EMS models.

## Data Availability

The data underlying this article will be shared on reasonable request to the corresponding author.
